# West Nile virus seroprevalence and associated risk factors among horses in Egypt

**DOI:** 10.1038/s41598-021-00449-6

**Published:** 2021-10-22

**Authors:** Abdelfattah Selim, Ameer Megahed, Sahar Kandeel, Abdulaziz Alouffi, Mashal M. Almutairi

**Affiliations:** 1 Department of Animal Medicine (Infectious Diseases), Faculty of Veterinary Medicine, Benha University, Moshtohor-Toukh, 13736 Kalyobiya Egypt; 2 Department of Animal Medicine (Internal Medicine), Faculty of Veterinary Medicine, Benha University, Moshtohor-Toukh, 13736 Kalyobiya Egypt; 3 Department of Veterinary Clinical Medicine, College of Veterinary Medicine, University of Illinois at Urbana-Champaign, Illinois, IL 61802 USA; 4 King Abdulaziz City for Science and Technology, Riyadh, 12354 Saudi Arabia; 5 Vaccines Research of Infectious Diseases, King Saud University, Riyadh, 11495 Saudi Arabia; 6 Department of Pharmacology and Toxicology, College of Pharmacy, King Saud University, Riyadh, Saudi Arabia

**Keywords:** Viral infection, Risk factors, Viral epidemiology

## Abstract

Determination of the seroprevalence and risk factors that are associated with West Nile virus (WNV) in horses is essential for adoption of effective prevention strategies. Our objective in this study, therefore, was to determine the seroprevalence and to identify the risk factors associated with WNV infection in the most densely horse-populated governorates in Egypt. A cross-sectional study was conducted in 2018 on 930 horses, which were distributed over five governorates in the Nile delta of Egypt. The horses, which were randomly selected, were serologically tested through use of an ID screen West Nile competition enzyme-linked immunosorbent assay (ELISA) to detect anti-WNV immunoglobulin G (IgG) and plaque reduction neutralization tests (PRNT; gold standard) to confirm the seropositive status of animals and to avoid cross reaction with other flavi-viruses. Four variables (geographical location, breed, sex and age) were considered in the risk analysis. Univariable and stepwise forward multivariable logistic regression methods were used for risk-factor analysis. The odds ratio (OR) was used as an approximate measure of relative risk. A total of 156 (16.8%; 95% confidence interval (CI) 14.4–19.2; *P* < 0.001) serum samples were found to be serologically positive for WNV. The highest seroprevalence rate was detected in horses of age ≥ 15 years (68.1%; 95% CI 49.8–72.4), stallions (26.4%; 95% CI 22.7–30.4), and those of mixed breed (21.5%; 95% CI 17.7–27.5). Horses older than 15 years were found to be at increased risk of WNV infection with OR = 4.3 (95% CI 3.0–6.2, *P* < 0.001) compared with horses aged under 2.5 years. Also, when all the risk factors were considered, stallions were more likely than mares to be WNV seropositive (OR = 2.4, 95% CI 1.6–3.7, *P* < 0.001), and of the breeds, mixed-breed (OR = 1.9, 95% CI 1.2–2.8, *P* = 0.005) and Arabian horses (OR = 1.9, 95% CI 1.2–2.8, *P* = 0.005) were more likely to be seropositive. Geographical location seemed to have no impact on the seroprevalence of exposure to WNV among these horses. Due to these findings, we strongly recommend intensive surveillance and implementation of effective control and prevention strategies against WNV, especially in stallion, mixed-breed horses with ages ≥ 15 years.

## Introduction

West Nile fever (WNF) is a zoonotic, widespread, vector-borne infection caused by West Nile virus (WNV), which belongs to the family *Flaviviridae*^1,2^. The disease is listed by the World Organization for Animal Health (OIE) as a notifiable disease, which means that animal owners and member countries are required to report its occurrence. West Nile virus is maintained in nature and is spread in an enzootic cycle between its biological vector (mosquitoes) and birds (primary host)^3^. In Egypt, *Culex antennatus* is the major mosquito species that is responsible for WNV transmission in the amplification cycle^3^. Around the world, more than 300 bird species may act as vertebrate hosts for WNV. Infected migratory birds are thought to play the major role in the spread of the virus to virus-free areas^4^. West Nile virus infects humans and horses through the bites of infected mosquitoes. Humans and horses are dead-end hosts that do not contribute to the spread or amplification of the virus^5,6^.

West Nile virus causes severe neuro-invasive illness in 1–10% of infected horses^7^. The typical clinical sign of infection is fever, which is followed by neurological signs such as ataxia, weakness of hind limbs, anorexia, depression, recumbency with an inability to rise, head pressing, signs of colic and even coma. Behaviour changes that range from aggression and hyperexcitability to somnolence may also be seen^8–11^.

WNV was first reported in 1937 in the West Nile region of Uganda. Since then the virus has become endemic in many African countries, as well as in the Middle East, Europe and Asia^12^. Recently, the disease has been reported among horses in the eastern and central regions of Saudi Arabia with a prevalence rate of 46.5%^13^. Since the 1960s, sporadic cases and outbreaks of WNV infection in both humans and horses have been reported in Europe. Surveys in parts of Europe and the Middle East have shown that nearly one-third of tested horses have been exposed to WNV, even though they show no clinical signs^7,14^. In the United States, WNV was first reported in crows in the eastern states, and it caused a severe epidemic of meningoencephalitis among people in New York City in 1999^15^. Later, the virus spread throughout Central America and Canada^4^. WNV was first recorded in the Mediterranean basin, including Egypt and Israel, in the early 1950s^3,16^. Several outbreaks among equine populations in the Mediterranean region sparked awareness of the importance of the disease and raised interest in research to predict where and when the virus would appear next^3,17^. However, few small-scale studies have been conducted to investigate the prevalence of WNV infection among horses in Egypt. They have reported seroprevalence that ranges from 20.7 to 25.5%^18,19^.

Definition of the risk factors that lead to infection with WNV is a key to implementation of successful prevention and control strategies, and helps animal owners to decide whether or not to vaccinate horses^20^. Animal-related factors such as age and breed have shown an association with the seroprevalence of exposure to WNV among horses^21^. However, the effect of these factors remains unclear and warrants further investigation. The main objective of this study was therefore to investigate the seroprevalence of exposure to WNV among horses that had been raised in the Nile delta of Egypt, as well as to determine the risk factors that were associated with exposure to WNV in these horses.

## Materials and methods

### Ethical approval

The study was performed in accordance with relevant guidelines and regulations of the Internal Ethics Review Committee of the Faculty of Veterinary Medicine, Benha University. Approval was obtained from the Faculty of Veterinary Medicine, University of Benha Animal Ethics Committee.

Blood was collected after receipt of each horse owner’s informed consent; we obtained statements that implied “informed consent” of owners.

### Study area

**Figure 1:**
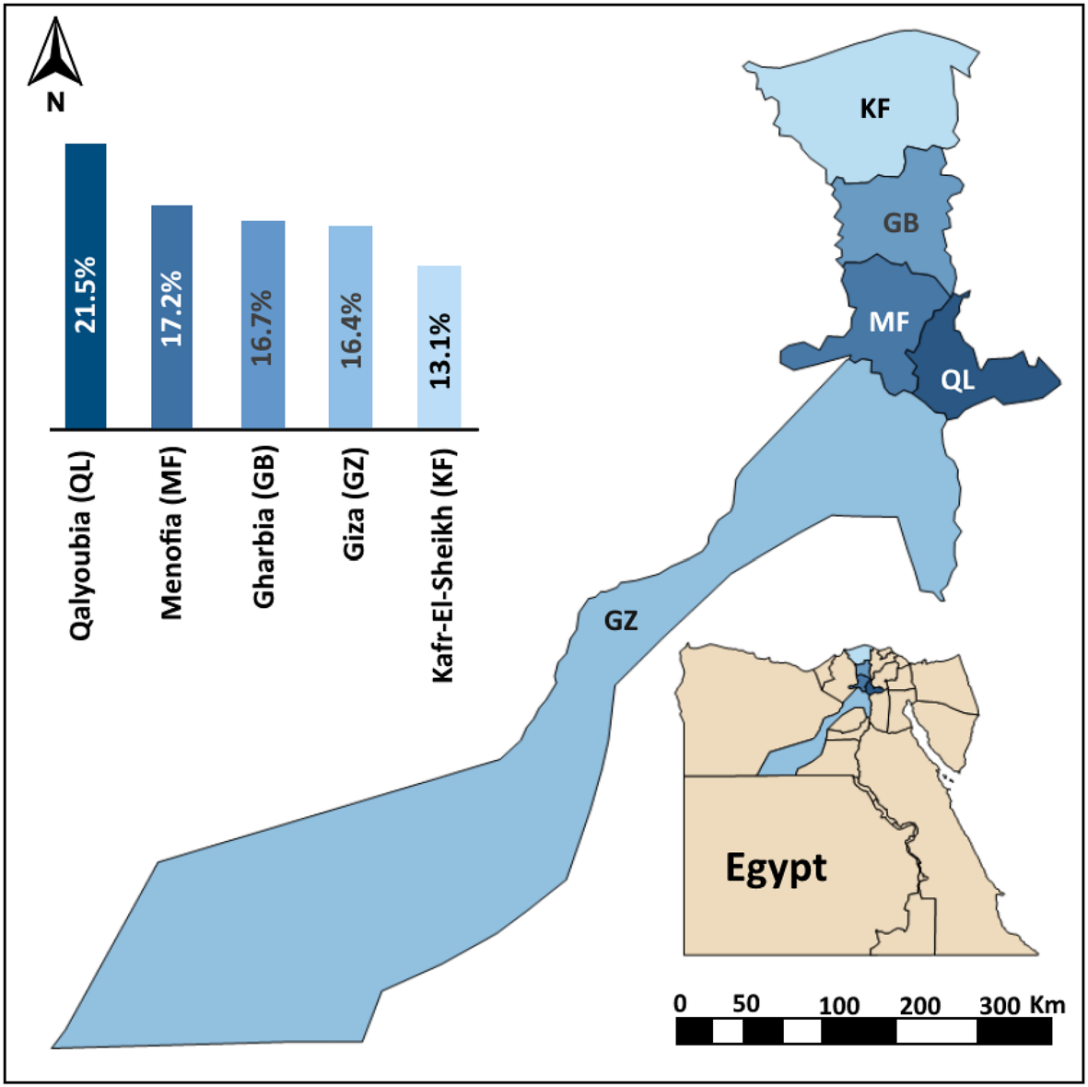
Distribution of West Nile virus infection (WNV) in horses of the Nile Delta of Egypt. The column graph showed the seroprevalence of WNV infection for each governorate.

This study was carried out in five governorates (Kafr Elsheikh (KF); Gharbia (GB); Menofia (MF); Qalyoubia (QL); and Giza (GZ)). These governorates are located in the northern part of Egypt (from 38° 18′ N to 30° 56′ E, 30° 52′ N to 31° 2′ E, 30° 31′ N to 30° 59′ E, 30° 25′ N to 31° 13′ E and 30° 01′ N to 31° 13′ E, respectively), as shown in Fig. 1. These governorates were selected as they were considered to be the most densely horse-populated areas of Egypt. The climate of these areas is hot in the summer (average 34 °C) and cool in the winter (average 10 °C), when the low temperatures are accompanied by rain. The climate of this region aids the multiplication and distribution of the vectors.

### Animals and sample collection

The sample size (930 horses) that was used for this study was calculated to be adequate based on a calculation that involved Cochran’s formula^22^, as follows:

$$n={Z}^{2}\frac{p(1-p)}{{e}^{2}}$$

in which n is the sample size, Z is the statistic corresponding to the level of confidence, *p* is expected prevalence, and *e* is the precision (corresponding to effect size). The level of confidence that was used in this study was 95%, the precision (*e*) was 5% and the expected prevalence was 20.7%, based on the prevalence that had been reported in an earlier study^18^.

Following a cross-sectional study design, 930 healthy horses were screened for the presence of immunoglobulin G (IgG) antibodies against WNV during the late spring and summer months of 2018. The horses were stratified by governorates according to the approximate number of horses that were understood to be in each governorate according to figures from the animal wealth development department of each governorate. Within governorates, the horses to be studied were selected randomly from different geographical locations. Most of the horses that were enrolled in this study had been raised to pull horse-drawn carriages or for horse-based tourism. Of the horses that had been raised on farms, either one or two horses were chosen randomly from each farm depending on the size of the farm. All horses had been raised in stables, whether on horse farms or by individual farmers. Horses were required to be resident in each locality at the time of sampling in order to exclude the potential of importing WNV from endemic areas.

Summaries of horse information were obtained from owners before blood was collected. Blood samples were randomly collected from 500 stallions, 300 mares and 130 geldings that ranged in age from 2 to 16 years and which had no history of vaccination or clinical signs related to WNV infection. Blood samples were collected from the jugular vein using 20G needles and 10 ml blood collection tubes. Serum was collected after centrifugation of the blood samples at 1300×*g*/min for 10 min. The serum samples were stored at − 20 °C until serological testing could be performed.

### Serological testing using ELISA

All serum samples were serologically tested for the presence of IgG antibodies against WNV using an ID screen West Nile competition enzyme-linked immunosorbent assay (ELISA) kit (IDVet, Montpellier, France) according to the manufacturer`s instructions. Infected horses retain a sufficient level of IgG antibodies for detection for one year after infection^23^. The optical density of samples was measured at 450 nm and the sample was considered positive if the sample/negative control percentage (S/N%) was ≤ 40% and negative if S/N% was > 50%. Additionally, the samples that were positive for WNV antibodies according to the ELISA were tested using plaque reduction neutralisation tests (PRNTs). These are considered the gold standard by the OIE to confirm their status^24^. PRNTs were performed using WNV (strain NY99-35261-11) and vero cells. Sera were tested using a starting dilution of 1:20. Titres were expressed as the reciprocal of serum dilutions that yielded a ≥ 90% reduction in the number of plaques (PRNT_90_).

### Statistical analysis

The seroprevalence of WNV infection was estimated with the exact binomial confidence intervals (CIs) of 95% by use of the PROC FREQ of SAS analytical procedure. The associations of WNV infection with different risk factors were evaluated using the Cochran-Armitage trend test, and the strength of associations was evaluated through phi and Cramer’s V value using the PROC FREQ of SAS analytical procedure. Correlation between the S/N% figures produced by ELISA and the 90% PRNT titres was evaluated by application of the Passing-Bablok regression plot. Univariable and stepwise forward multivariable logistic regression were used to identify the most important risk factor(s) that were associated with WNV. The predicted probability curves were created using the logistic regression model-predicted probabilities. For stepwise forward multivariable logistic regression, the *P*-values for entry into or removal from the logistic regression models were < 0.05. The logistic model, fitted with WNV infection as the outcome variable (present: 1, absent: 0), included fixed effects of the risk factors of breed (three levels: Arabian, thoroughbred, and mixed), sex (three levels: stallion, mare and gelding), age (five levels: < 2.5, 2.5–< 5, 5–< 10, 10–< 15, ≥ 15 years), and geographical location (five levels: KF, GB, MF, QL and GZ). The stepwise elimination process was stopped once all remaining variables were found to contribute significantly (*P* < 0.05) to the model. The fit of the multivariable logistic regression model was assessed through application of the Hosmer–Lemeshow goodness-of-fit test.

A logistic regression model predicts the log odds (logit) for outcome as an additive function of the risk factors. The odds ratio (OR) was used as an approximate measure of relative risk (the likelihood of having a positive result for IgG ELISA in an animal with a given risk factor compared with an animal without the risk factor). The 95% CIs for OR estimates were obtained as described by Lemeshow and Hosmer^25^. ORs of > 1 indicate an increased risk of the outcome (seropositivity to WNV) with increasing value of risk factor, and ORs of < 1 indicate a decreased risk of the outcome (seropositivity to WNV) with increasing value of the risk factor. Statistical analyses were performed using SAS 9.4 (SAS Inc., Cary, NC).

## Results

### Seroprevalence of WNV

The seroprevalence of WNV infection was determined in 930 serum samples that were obtained from three different breeds of horses (Arabian, thoroughbred, and mixed) that were located in five governorates in the Nile delta of Egypt (KF, GB, MF, QL and GZ) and were of ages that ranged from 2 to 16 years. The distribution of horses based on the risk factors is illustrated in Table 1.

**Table 1:** Descriptive analysis of variables used to predict the seroprevalence of West Nile virus infection in equine of the Nile Delta of Egypt.

Variable	Category	No. of horse	Distribution (%)
Governorates	Kafr-El-Sheikh	160	17.2
	Gharbia	60	6.5
	Menofia	250	26.9
	Qalyoubia	130	14.0
	Giza	330	35.5
Breed	Arabian	300	32.3
	Thoroughbred	351	37.7
	Mixed	279	30.0
Sex	Stallion	500	53.8
	Mare	300	32.3
	Gelding	130	14.9
Age (years)	< 2.5	29	3.1
	2.5–< 5.0	167	18.0
	5.0–< 10	463	49.8
	10.0–< 15.0	203	21.8
	≥ 15.0	68	7.31

**Figure 2:**
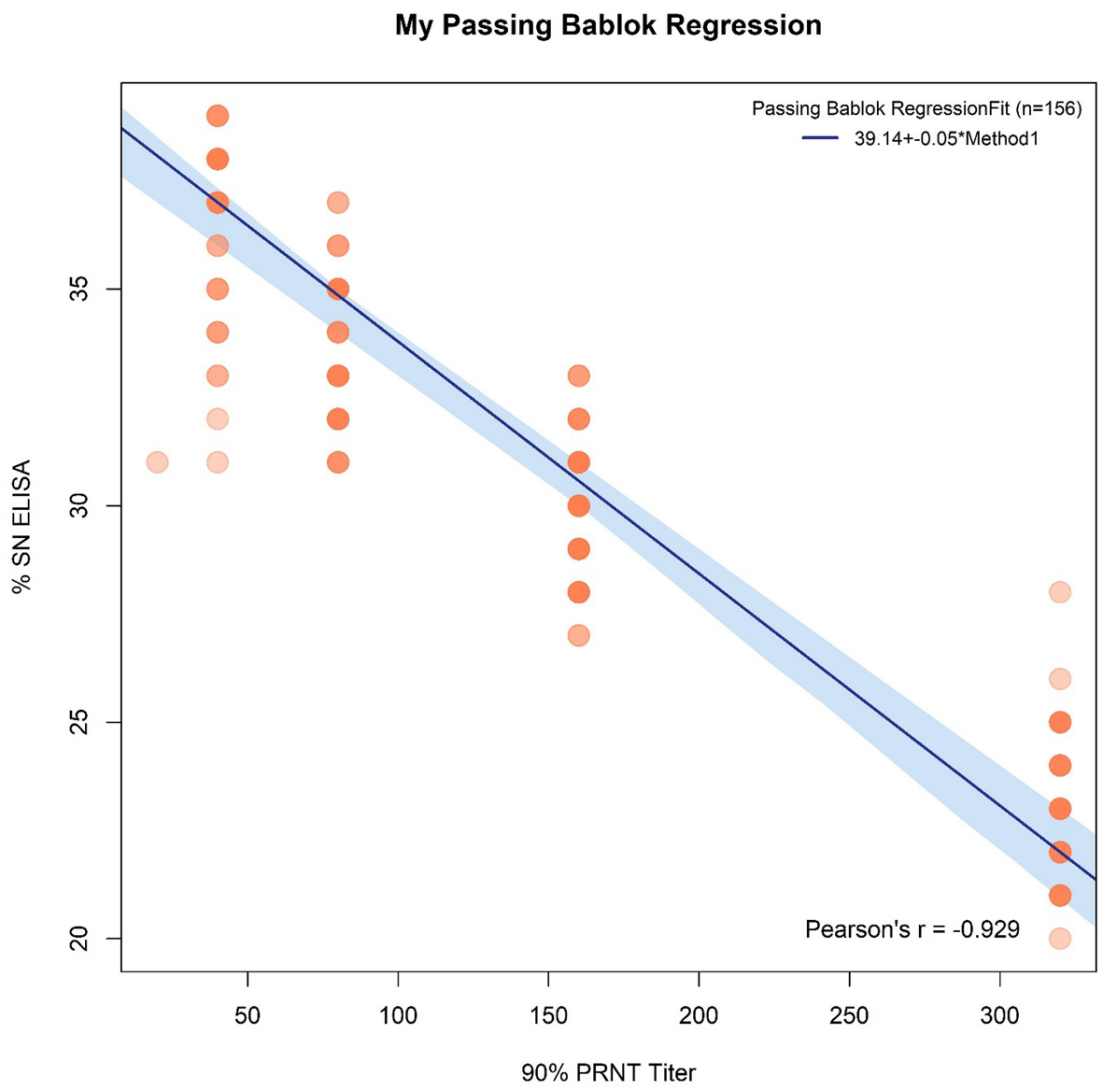
Correlation between SN% of ELISA and 90% PRNT titre.

Of 930 tested horse serum samples, 156 sera (16.8%) showed antibodies by indirect ELISA (95% CI 14.4–19.2; P < 0.001). The same horses that tested positive by ELISA (n = 156) were positive with the PRNT. In addition, the results revealed strong correlation between the S/N% of ELISA and 90% PRNT titre (Pearson's correlation coefficient = − 0.93; P < 0.001 and Spearman's rank correlation rho = − 0.91; P < 0.001). These results are shown in Fig. 2.

The results of univariable logistic regression showed that the seroprevalence of WNV infection differed non-significantly (*P* = 0.768) between the localities that were investigated. However, horses from the QL governorate showed the highest seroprevalence of WNV (21.5%), followed by MF (17.2%), GB (16.7%), GZ (16.4%) and KF (13.1%), as shown in Table 2 and Fig. 1. In contrast, the distribution of WNV positivity was dependent on age (*P* < 0.001), sex (*P* < 0.001), and breed of the horse (*P* < 0.01; Table 2). The results of this study showed a moderate association between the seroprevalence of exposure to WNV infection and age (phi coefficient and Cramer’s V = 0.56), and weak association with sex (0.28). However, phi coefficients/Cramer’s Vs of 0.10 and 0.06 indicated that there were no associations between the seroprevalence of WNV infection and breed and geographical location, respectively.

**Table 2:** Univariable logistic regression analysis of the association of horse-level West Nile virus infection with different risk factors in the Nile Delta of Egypt.

Variable	Category	No. of horses	No. positive	Prevalence (%)	*P* value
Governorates	Kafr-El-Sheikh	160	21	13.1	0.768
	Gharbia	60	10	16.7	
	Menofia	250	43	17.2	
	Qalyoubia	130	28	21.5	
	Giza	330	54	16.4	
Breed	Arabian	300	40	13.3	0.005
	Thoroughbred	351	54	15.4	
	Mixed	279	62	22.2	
Sex	Stallion	500	132	26.4	< 0.001
	Mare	300	12	4.0	
	Gelding	130	12	9.2	
Age (year)	< 2.5	29	0	0.0	< 0.001
	2.5–< 5.0	167	8	4.8	
	5.0–< 10.0	463	31	6.7	
	10.0–< 15.0	203	75	37.0	
	≥ 15.0	68	42	61.8	

**Figure 3:**
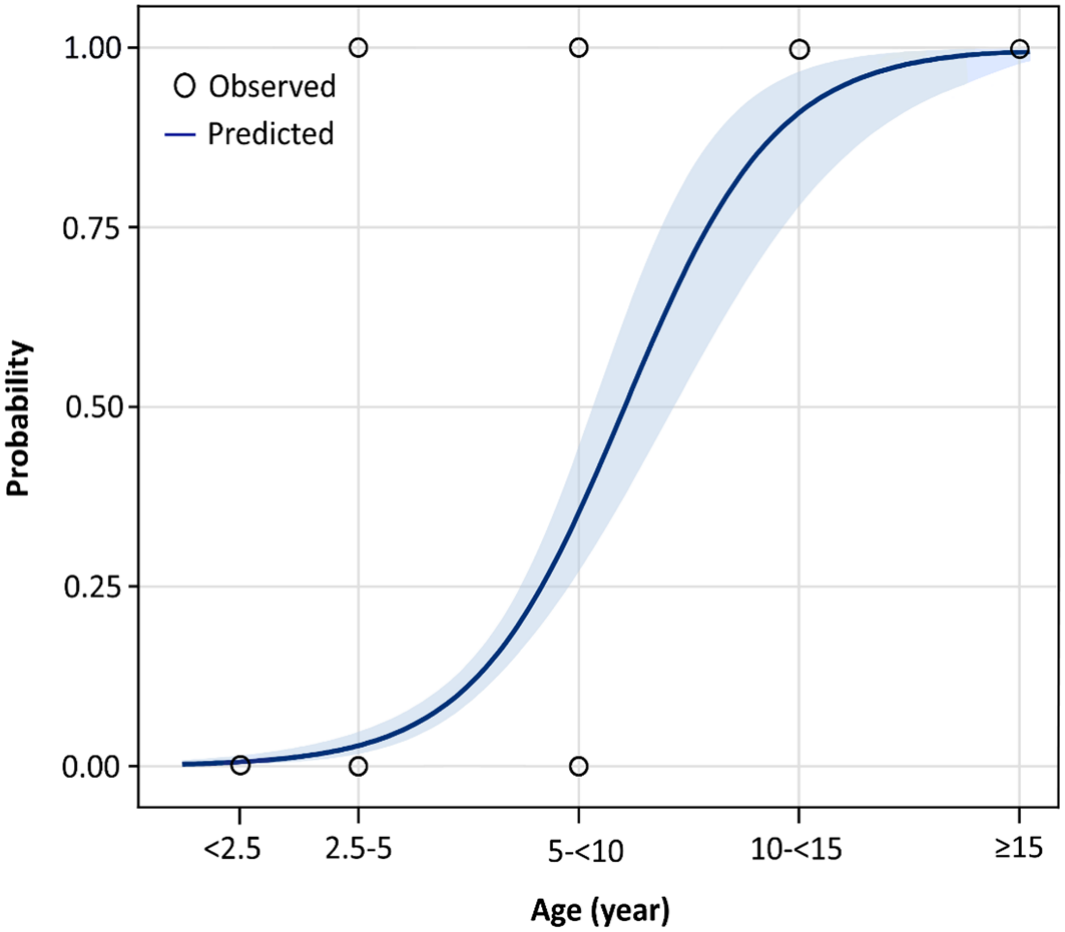
Probability plot for the ability of age to predict the seroprevalence of West Nile virus (WNV) infection in the Nile Delta horses in Egypt. The curve shows the likely probability of seropositive WNV associated with age, with the 95% shaded blue confidence interval.

The final model of stepwise logistic regression indicated that age, sex and breed were significant risk factors for seropositivity to WNV infection (Table 3). Geriatric horses (aged over 15 years) were 4.3 (95% CI 3.0–6.2, *P* < 0.001) times more likely to show WNV infection than horses aged under 2.5 years; the horses that were aged over 15 years showed a 99.0% probability of being seropositive for WNV infection. Interestingly, the probability of risk increased with age, as shown in Fig. 3. Stallions were more likely to be WNV seropositive than mares (OR = 2.4, 95% CI 1.6–3.7, *P* < 0.001), whereas geldings were not significantly more or less likely to be seropositive than mares. Mixed-breed horses were also found to be at higher risk of exposure to WNV (OR = 1.9, 95% CI 1.2–2.8, *P* = 0.005) than Arabian horses, whereas thoroughbred horses were not significantly more or less likely to be seropositive than Arabian horses. However, geographical location showed no impact on the seroprevalence of WNV infection (OR = 0.9, 95% CI 0.8–1.1, *P* = 0.7698).

**Table 3:** Multiple stepwise logistic regression analysis of potential risk factors associate with West Nile virus seropositivity in horse in the Nile Delta of Egypt.

Variable	Categories	*β*	SE	*P* value	OR	95% CI_OR_
Intercept		− 8.39	0.98	< 0.001	–	–
Age	< 2.5	Reference				
	2.5–< 5.0	0.94	0.17	0.303	1.7	0.9–3.2
	5.0–< 10.0	0.84	0.14	0.012	2.3	1.3–6.6
	10.0–< 15.0	1.29	0.23	< 0.001	3.6	2.1–5.4
	≥ 15.0	1.46	0.19	< 0.001	4.3	3.0–6.2
Sex	Mare	Reference				
	Stallion	0.94	0.22	< 0.001	2.4	1.6–3.7
	Gelding	0.80	0.34	0.109	1.3	0.8–4.2
Breed	Arabian	Reference				
	Thoroughbred	0.83	0.41	0. 204	1.4	0.9–2.3
	Mixed	0.89	0.21	0.005	1.9	1.2–2.8

## Discussion

WNV is a mosquito-borne pathogen of global public health importance. With the rapid global spread of WNV and its endemic status in many places, WNV might spread into new geographical areas of Egypt. To the best of our knowledge, this is the first large-scale study to have investigated the seroprevalence of WNV among horses and the associated risk factors for infection of horses by WNV in Egypt. Therefore, the results of this study provide contemporary information about WNV seroprevalence and associated risk factors that can be used as a guide for the introduction of effective prevention strategies.

The major advantages of the present study were: (1) the broad context of many governorates, which places this study among the few that have examined the seroprevalence of WNV antibodies in horses across the most densely equine-populated areas in Egypt; and (2) the large number of horses that were investigated, which confers a robustness of association between variables compared with earlier Egyptian studies. The main finding of this study was that stallions of mixed breed and of ages ≥ 15 years were the most exposed to WNV in Egypt compared with other sexes, breeds and ages of horses.

WNV has been circulating in Egypt since 1951^16^. Most of the earlier studies that were conducted in the country were focused on the investigation of WNV epidemiology in humans^16,26,27^. Recently, increased attention has been paid to the epidemiological status of WNV among horses in Egypt^18^. Overall, antibodies against WNV were detected in 156 of 930 (16.8%) examined horses by ELISA and PRNT tests. Several studies have reported lack of specificity of the ELISA test in the detection of anti-WNV antibodies due to cross-reaction with other flaviviruses^28,29^. Therefore, the positive samples were confirmed by PRNT, which showed the same results as the ELISA tests. These results confirm that circulation of WNV occurs among Egyptian horses.

The reported seroprevalence rate concurs with other rates that have been reported for different countries: 15.08% in Poland^30^ and 15% in Portugal^31^. However, this rate is lower than that reported in countries that surround Egypt. The prevalence of exposure to WNV among horses in Israel has been reported to be 39%^32^; 24.9% in Jordan^33^; 26.8% in Algeria^34^; 31.1% in Morocco^35^; 31.6% in Turkey^36^; and 68.7% in Senegal^34^. The low level of seroprevalence of WNV that is reported in this study might be because there are few horse farms in Egypt and therefore most of the horses that were investigated in this study were individually raised. This may have decreased the chances of exposure of the horses to the virus. Additionally, one of the main factors that affects the spread of WNV infection is the environment^26^. High temperatures (above 30 °C), which are found in the Nile delta of Egypt, particularly in summer, and low amounts of rainfall during winter and spring have been associated with a reduction in mosquito activity and with limited growth in the numbers of mosquitoes^37–41^. Consequently, the spread of WNV among horses is decreased because the propagation of WNV in mosquitoes is temperature-dependent^42^. A positive association has been reported between levels of WNV in mosquitoes and occurrence of WNV epidemics, and the previous year’s precipitation figures^32,43–45^. Other factors such as humidity, presence of local and migratory birds’ nesting sites, and the vegetation index also contribute significantly to the propagation of WNV^46^. Furthermore, in our study the majority of samples were collected during the early summer, whereas it is known that most WNV infection usually occurs in late summer and the autumn^33^. The difference between seroprevalence rates of WNV that were reported in this study and those that were reported in the earlier study might also be due in part to different sampling strategies^47^.

The results of the current study showed that the risk of exposure to WNV increased markedly with the horses’ ages, which suggested age-related vulnerability to WNV infection. This result is consistent with those of earlier studies^21,48,49^, yet there are other studies that did not show any association between the seropositivity for exposure to WNV and age^17,30,33^. It has been reported that older affected horses are more likely to die from WNV infection than younger animals^50–52^. The reason behind this positive relationship between seropositivity for exposure to WNV and age is unknown, but it might be attributable to cumulative exposure over time.

The second risk factor for exposure to WNV that was reported in this study was the sex of the animals. Our findings were in accordance with earlier findings^50^, which found a higher seroprevalence for exposure to WNV among stallions than among mares or geldings. The reason for this result is unclear, but the difference may be due to culture or management; stallions are preferred for pulling large wagons and carriages, and these occupations place them at greater risk of mosquito bites than do other tasks.

Intriguingly, the mixed-breed horses showed higher seroprevalence of WNV than the Arabian and thoroughbred horses, which was in agreement with the results of an earlier study^33^. However, another study reported no effect of breed on the seroprevalence of exposure to WNV in horses^30^. The reasons for the higher WNV seroprevalence that was found in our study among mixed-breed horses, compared with other breeds, is unknown, but it could be due to different management methods. Pure-bred horses are looked after more carefully than mixed breeds and are housed in stables, whereas mixed breeds are housed outdoors, which makes them vulnerable to attack by mosquitoes^32^. The results of this study showed low phi coefficient and Cramer’s V values regarding the association between WNV exposure and breed; however, breed was a significant predictor of seroprevalence of WNV infection. This might be because of the sample size effect^53–55^.

The main limitation of the present study was that, because it was a cross-sectional study, it could not be used to provide evidence of a cause-and-effect relationship. Longitudinal studies that involve larger sample sizes and broader geographical representation than this study are required to verify the associations that were found in this study. Other limitations were that the IgG ELISA tests showed cross-reactivity among related flaviviruses and the PRNTs showed lower sensitivity than the ELISA tests^7^; therefore, new technologies are required as alternatives to the usual serological tests.

## Conclusion

The results of the present study confirm the presence of WNV infection among horses in Egypt and provide updated information concerning the geographical areas that are affected by the virus. Our findings indicate that intensive surveillance and implementation of effective control and prevention of WNV infection are urgently required.
